# Supplementary figures and images for: Distinct mechanisms underlie H2O2 sensing in C. elegans head and tail
Source: PLoS One. 2022 Sep 29;17(9):e0274226. doi: 10.1371/journal.pone.0274226 (PMC9521893; doi:10.1371/journal.pone.0274226)

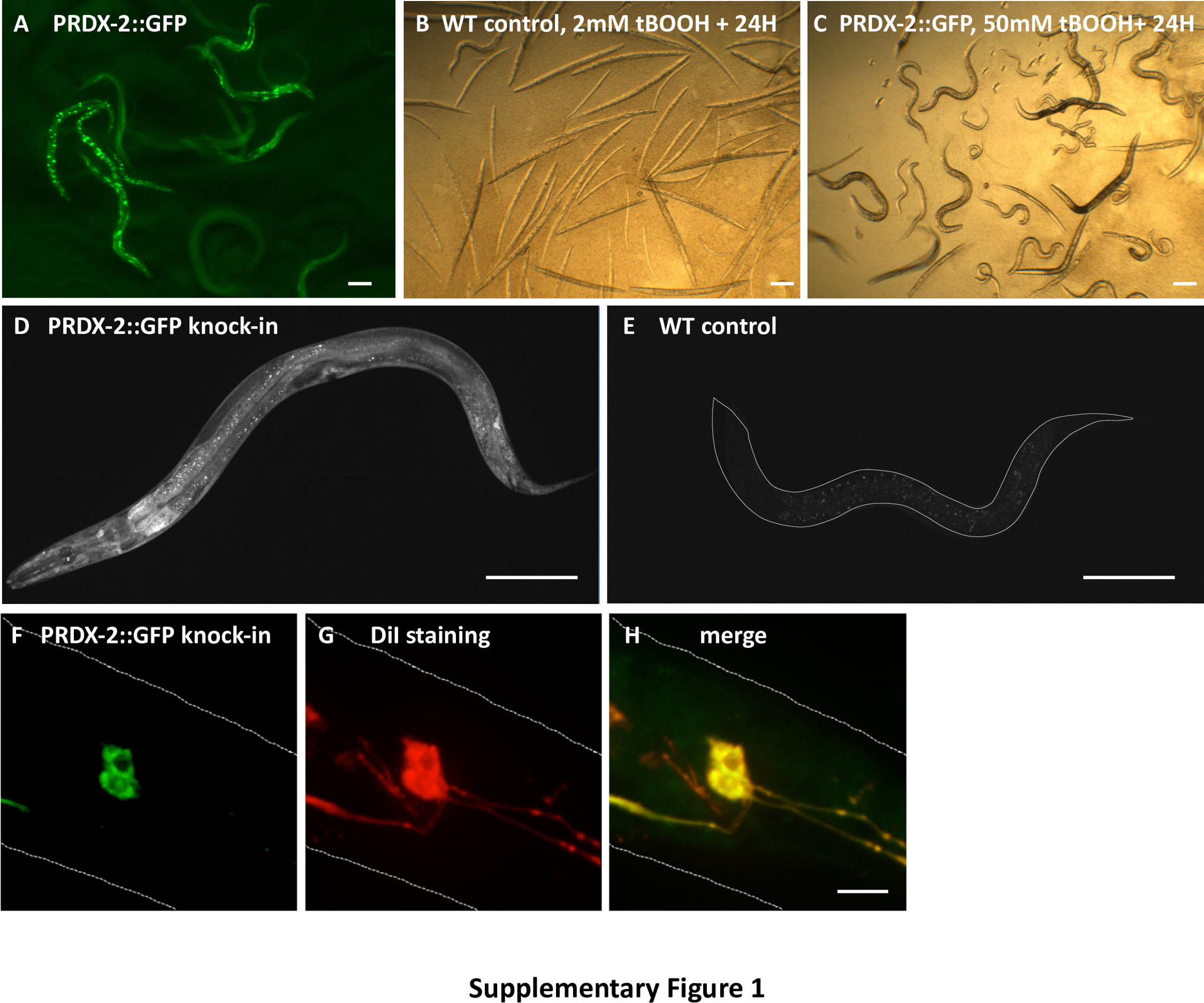

Supplement: S1 Fig — (A) Low magnification fluorescent image of untreated animals from the CTD1051.3 line showing that transgenics express aggregates of PRDX-2::GFP, a hallmark of overexpression of the fusion protein. (B,C) Low magnification images of animals treated in flat bottom wells imaged after 24h of treatment in the potent oxidative stress inducing agent tBOOH. In the wild-type control well (A), almost all animals are all dead in 2mM tBOOH, appearing as rods, while CTD1051.3 transgenics survive a 25X higher dose of the drug, indicating their much stronger resistance to oxidative stress. (D) Representative image of an animal of the PRDX-2::GFP knock-in line; a wild-type control imaged with the same settings is shown (E), delineated by a dotted line. Bar, 100μm. (F-H) Confocal projections showing the tail region (dotted contours) of a PRDX-2::GFP transgenic animal stained with the lipophilic orange-red dye DiI (see https://www.wormatlas.org/EMmethods/DiIDiO.htm), imaged in green (F) and red channels (G). The overlay (H) shows that PRDX-2::GFP tail neurons are stained by the dye, establishing their identity as phasmid sensory neurons (PHA/PHB). Bar, 10μm. (TIF) [file pone.0274226.s001.tif]

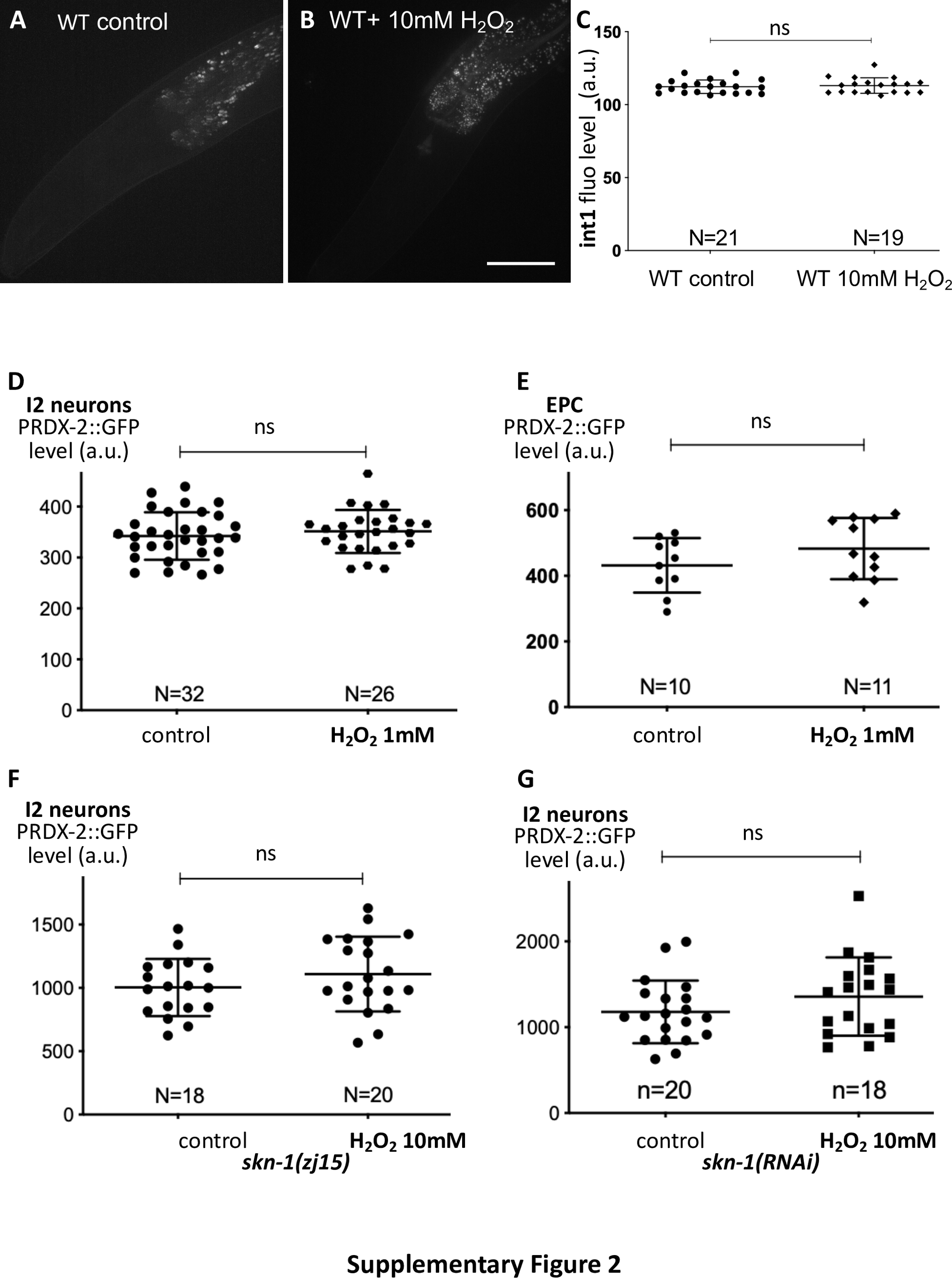

Supplement: S2 Fig — (A,B) Wild-type controls do not show a higher gut autofluorescence after H2O2 treatment, as illustrated by the int1 anterior gut cell fluorescence quantification (C). (D,E) Quantification of the PRDX-2::GFP fluorescence level in I2 neurons and in the excretory pore cell (EPC) in controls after 1mM-H2O2 treatment. (F,G) Quantification of I2 neurons’ PRDX-2::GFP fluorescence level in skn-1(zj15) mutants and in skn-1(RNAi) animals upon a 10mM-H2O2 treatment. Means are shown and error bars represent SD; ns, not significant, p>0.05 (t test or Mann-Whitney test). Scale bar, 50μm. (TIF) [file pone.0274226.s002.tif]

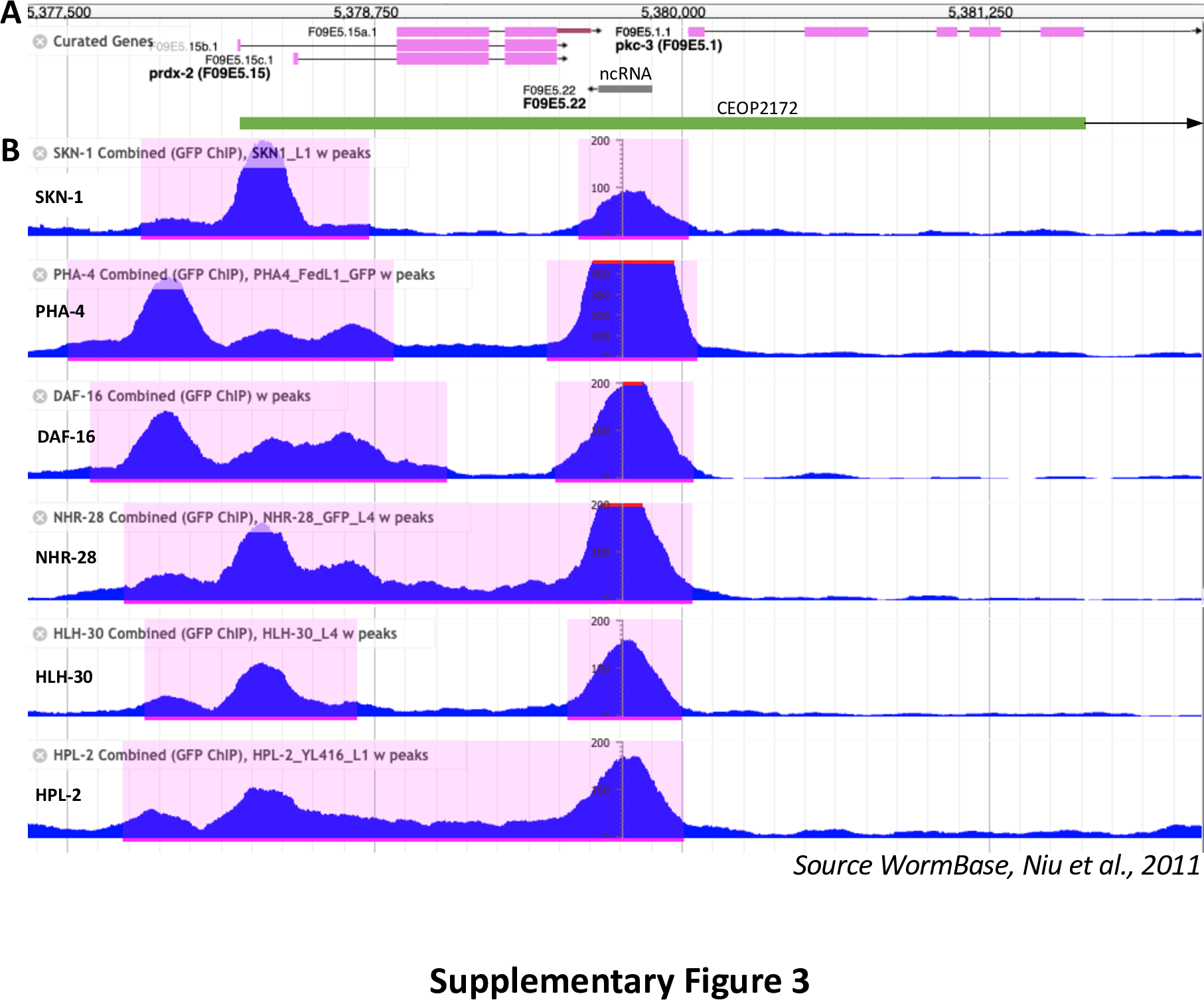

Supplement: S3 Fig — Genomic organization of the prdx-2 locus and presumptive regulation by SKN-1 and additional transcription factors. (A) Genome Browser screenshot from WormBase (release WS283, JBrowse II), showing the gene organization on chromosome II, at the indicated coordinates (top), and the peaks detected by ChIP-seq using an anti-GFP antibody [42] in prdx-2 and pkc-3 promoters, in GFP-tagged transgenic lines of the indicated transcription factors (B). Note the co-regulation of prdx-2 and pkc-3, which are organized in an operon, illustrated by the green bar. (TIF) [file pone.0274226.s003.tif]

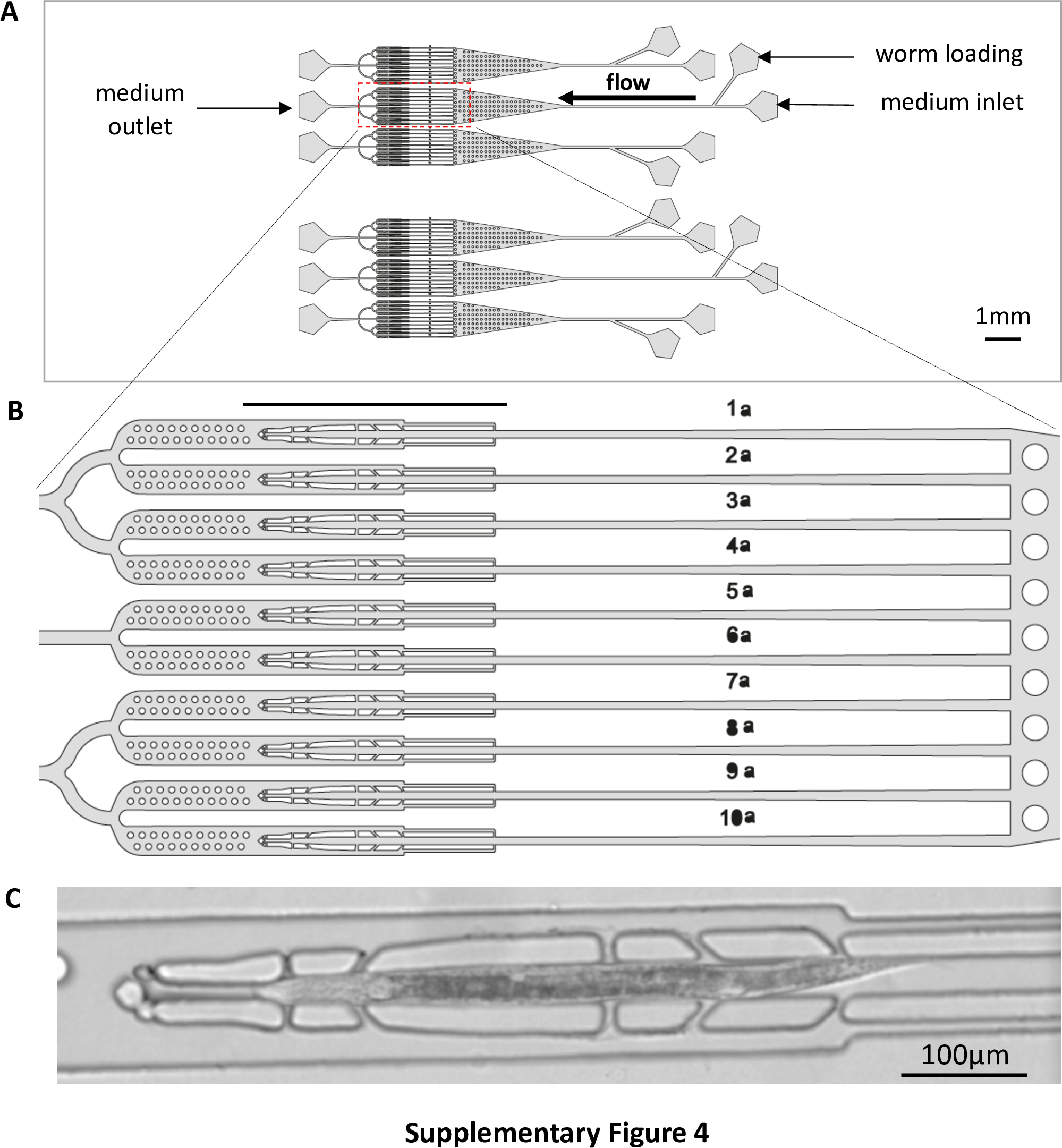

Supplement: S4 Fig — (A) Global view of the microfluidic chip used in all neuron response experiments, showing the 6 independent series of 10 worm traps. The original file (Autocad format) is available at https://github.com/gcharvin/viewworm. (B) Magnification of the worm trap area showing the 10 individual channels (corresponding to the red box in A). Scale bar, 1mm. (C) DIC image of an animal trapped (anterior to the left). Occasionally animals were trapped in the opposite orientation, but this did not affect the neuronal response. Scale bar, 100μm. (TIF) [file pone.0274226.s004.tif]

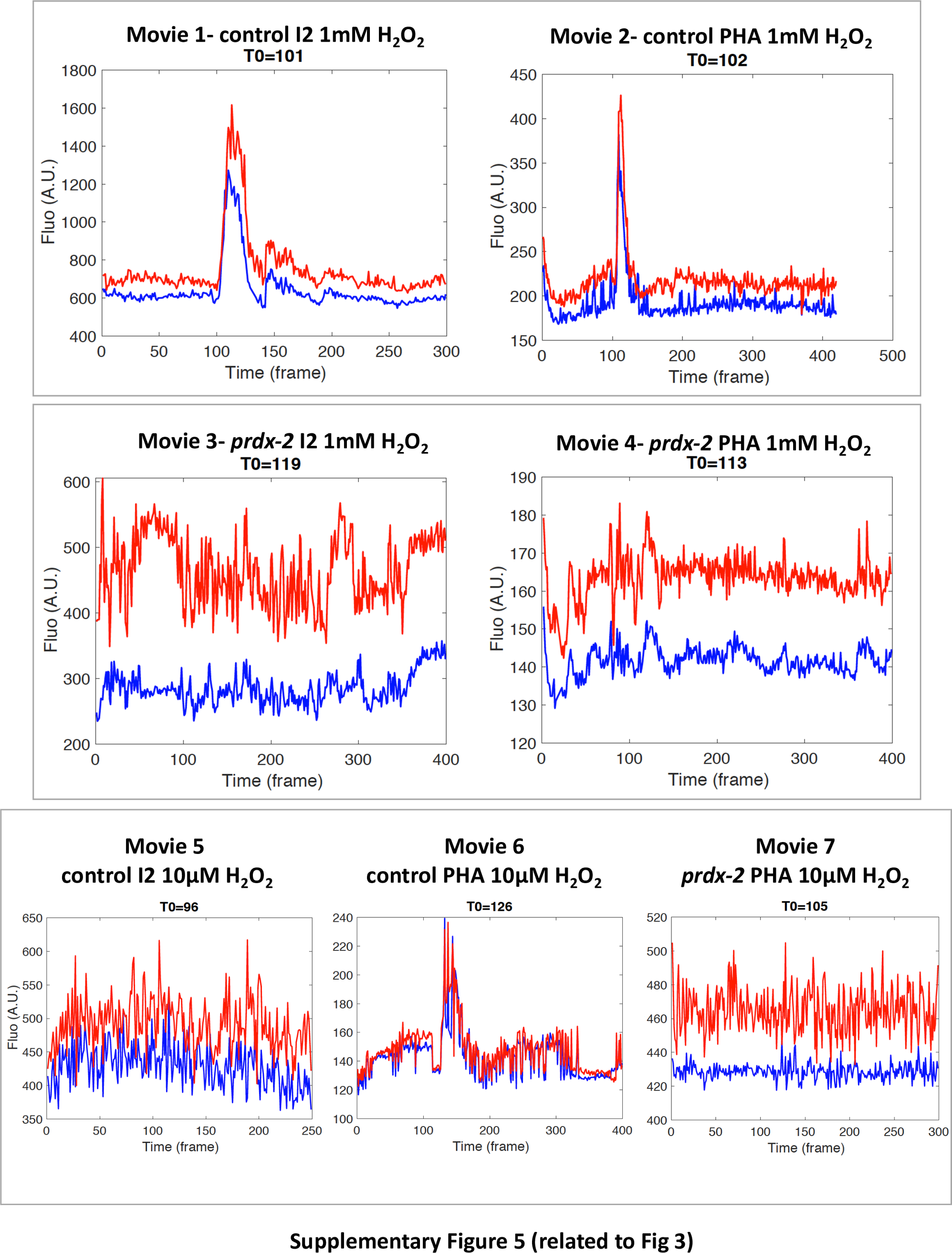

Supplement: S5 Fig — The curves represent the mean GCaMP3 intensity raw value over time (1 frame = 2sec) quantified in I2 and PHA neurons in control and prdx-2 mutants corresponding movies (S1–S7 Movies), upon a 1mM or a 10μM H2O2 exposure. T0 indicate the time point at which the H2O2 treatment has been applied during 100 frames. Red and blue colors represent left and right I2 and PHA neurons (ie I2L/R and PHAL/R). Note that the colors have been changed in normalized average curves shown in Fig 3 and in related S6 Fig. (TIF) [file pone.0274226.s005.tif]

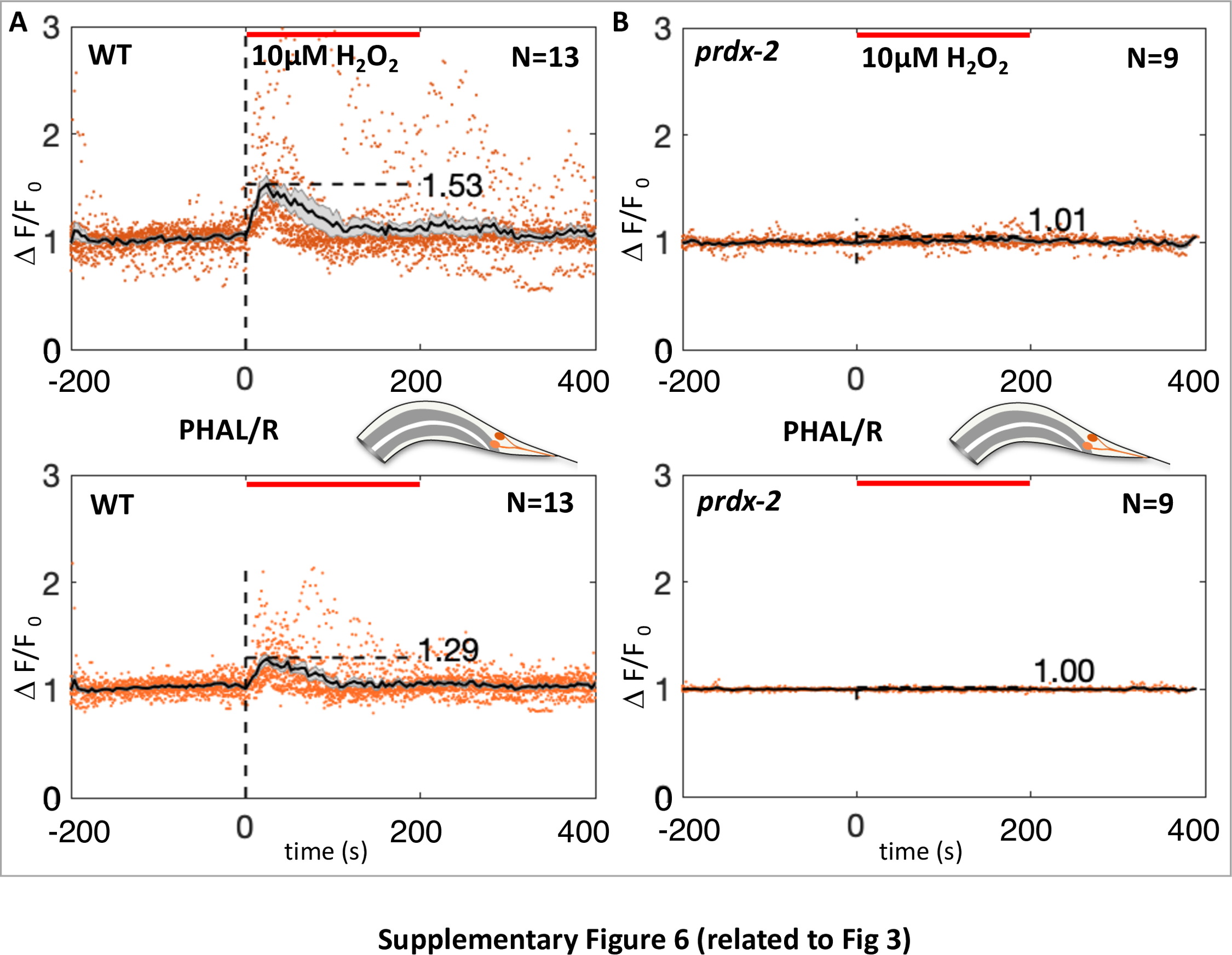

Supplement: S6 Fig — (A-D) Average curves showing the normalized calcium response to 10μM H2O2 measured over time (in seconds) using the GCaMP3 sensor in PHA left and right neurons (top and bottom curves) in prdx-2(gk169) mutants (B) and in wild-type controls (A). N, number of movies analyzed for each genotype. See S6–S7 Movies and related S5 Fig. (TIF) [file pone.0274226.s006.tif]

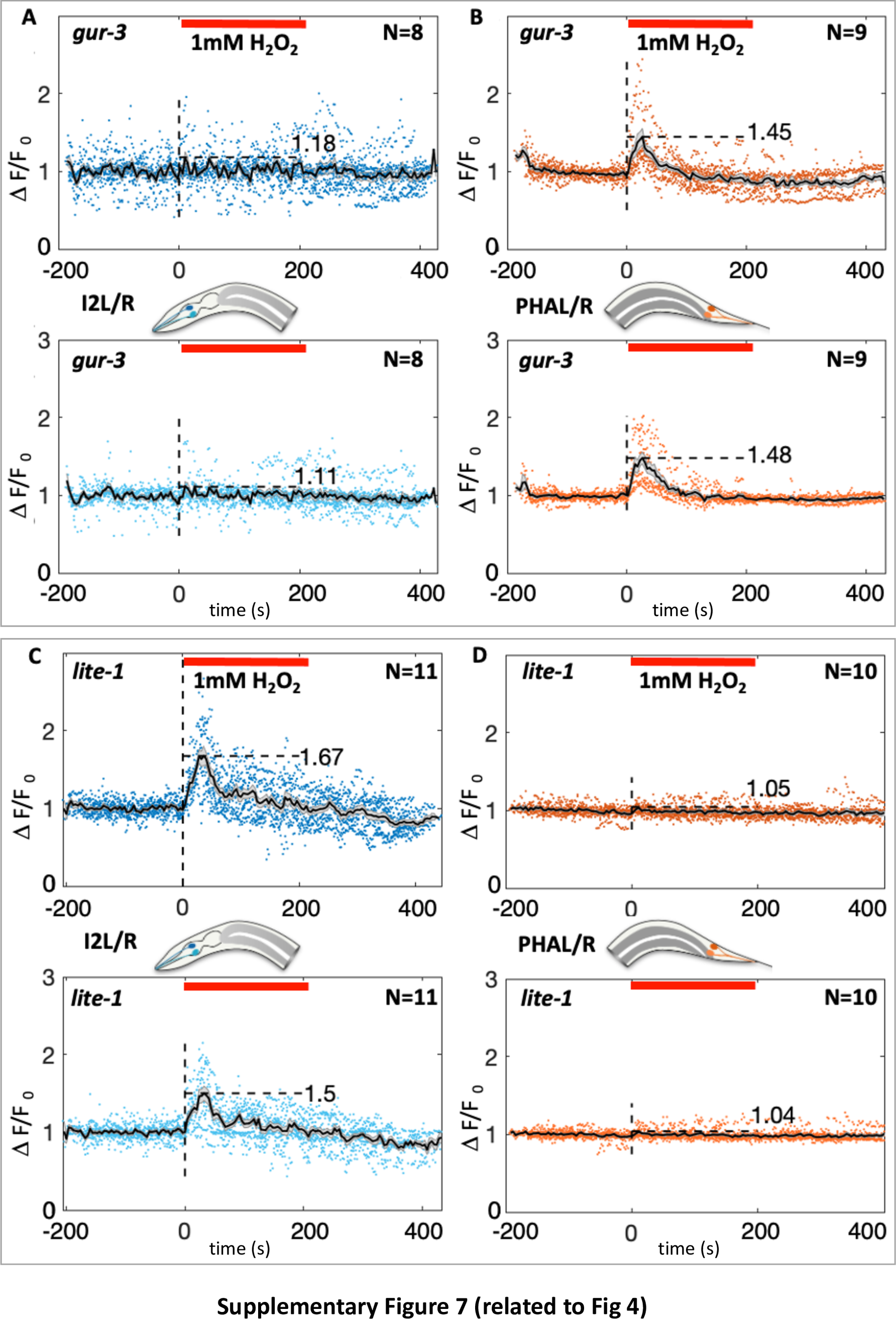

Supplement: S7 Fig — (A-D) Average curves showing the normalized calcium response to 1mM H2O2 measured over time (indicated in seconds) using the GCaMP3 sensor in I2 and PHA left and right neurons (top and bottom curves) in gur-3(ok2245) (A,B) and lite-1(ce314) mutants (C,D). N, number of movies analyzed for each genotype. See S8–S11 Movies and related S8 Fig. (TIF) [file pone.0274226.s007.tif]

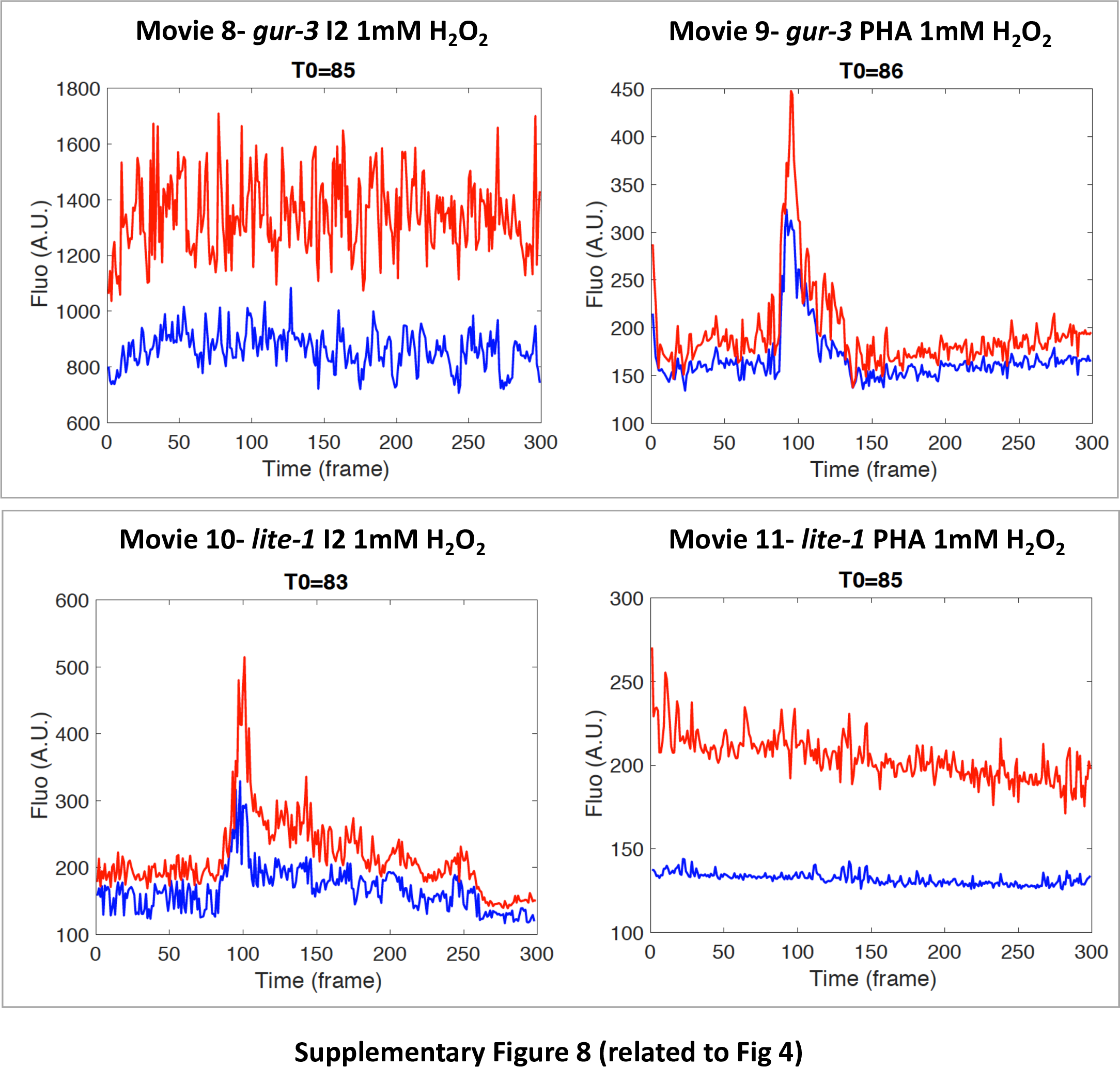

Supplement: S8 Fig — The curves represent the mean GCaMP3 intensity raw value over time (1 frame = 2sec) quantified in I2 and PHA neurons in gur-3 and lite-1 mutants upon a 1mM H2O2 exposure (starting at T0 and lasting 100 frames), in corresponding movies (S8–S11 Movies). Red and blue colors show left and right neurons for I2 (left panel) and PHA (right panel) neurons (ie I2L/R and PHAL/R). Note the reciprocal phenotypes observed in both mutants. Colors have been changed in the related normalized average curves shown in S7 Fig. (TIF) [file pone.0274226.s008.tif]

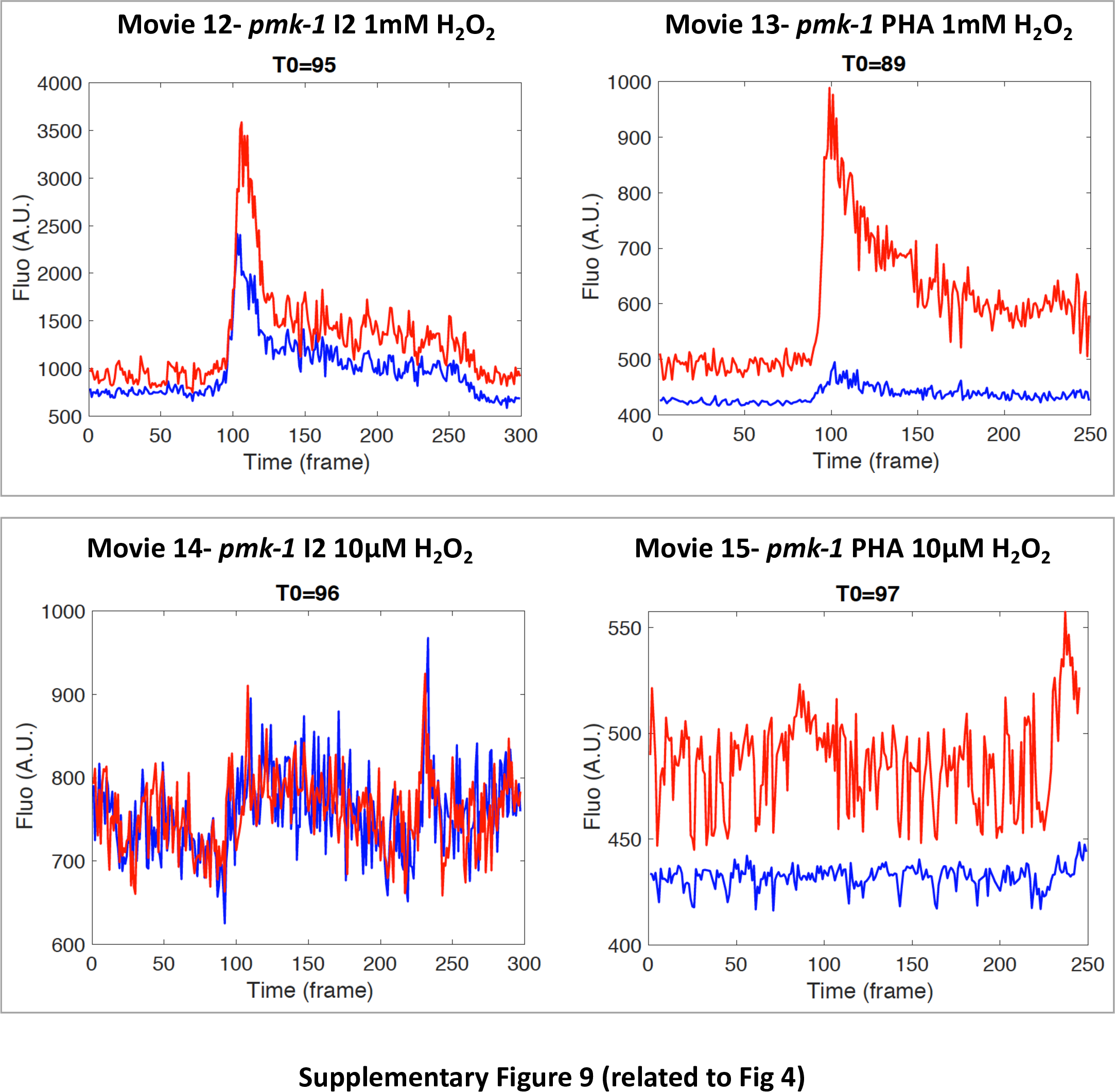

Supplement: S9 Fig — The curves represent the mean GCaMP3 intensity raw value over time (1 frame = 2sec) quantified in I2 and PHA neurons in pmk-1 mutants upon a 1mM or a 10μM H2O2 stimulation (starting at T0 and lasting 100 frames), in corresponding movies (S12–S15 Movies). Red and blue color indicate I2L/R neurons (left panel) and PHAL/R neurons (right panel). (TIF) [file pone.0274226.s009.tif]

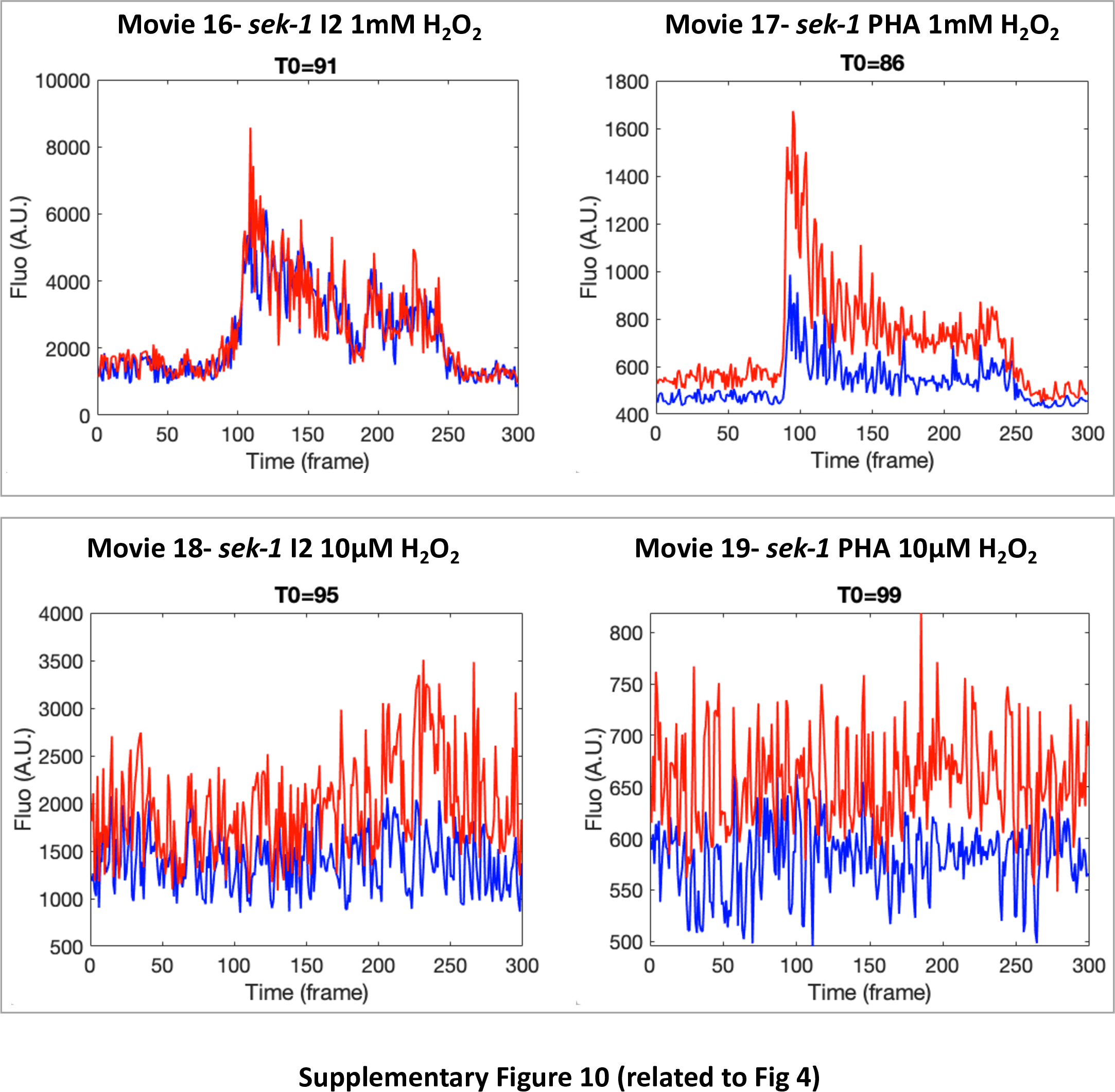

Supplement: S10 Fig — The curves represent the mean GCaMP3 intensity raw value over time (1 frame = 2sec) quantified in I2 and PHA neurons in sek-1 mutants upon a 1mM or a 10μM H2O2 stimulation (starting at T0 and lasting 100 frames), in corresponding movies (S16–S19 Movies). Red and blue color indicate left and right I2 (left panel) and PHA (right panel) neurons. (TIF) [file pone.0274226.s010.tif]

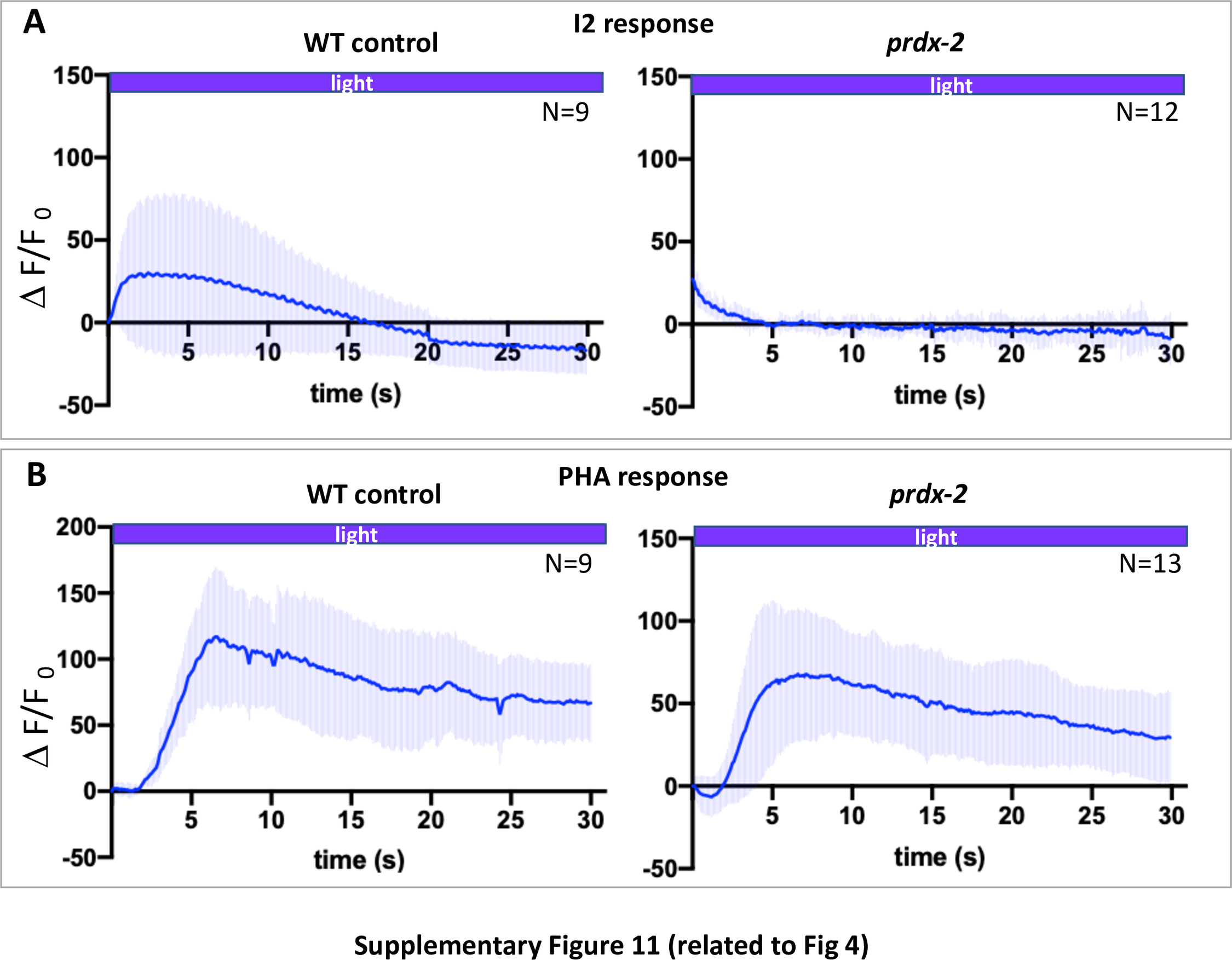

Supplement: S11 Fig — Average curves showing the normalized calcium response to blue light over time (in seconds) using the GCaMP3 sensor, measured in the soma of I2 (A) and PHA neurons (B) in wild-type controls and in prdx-2(gk169) mutants. N, number of movies analyzed for each genotype. Error bars represent SD. While I2 neurons fail to respond to light in prdx-2 mutants, PHA neurons do respond, albeit with a lower intensity peak than in controls. See related S20–S24 Movies. (TIF) [file pone.0274226.s011.tif]

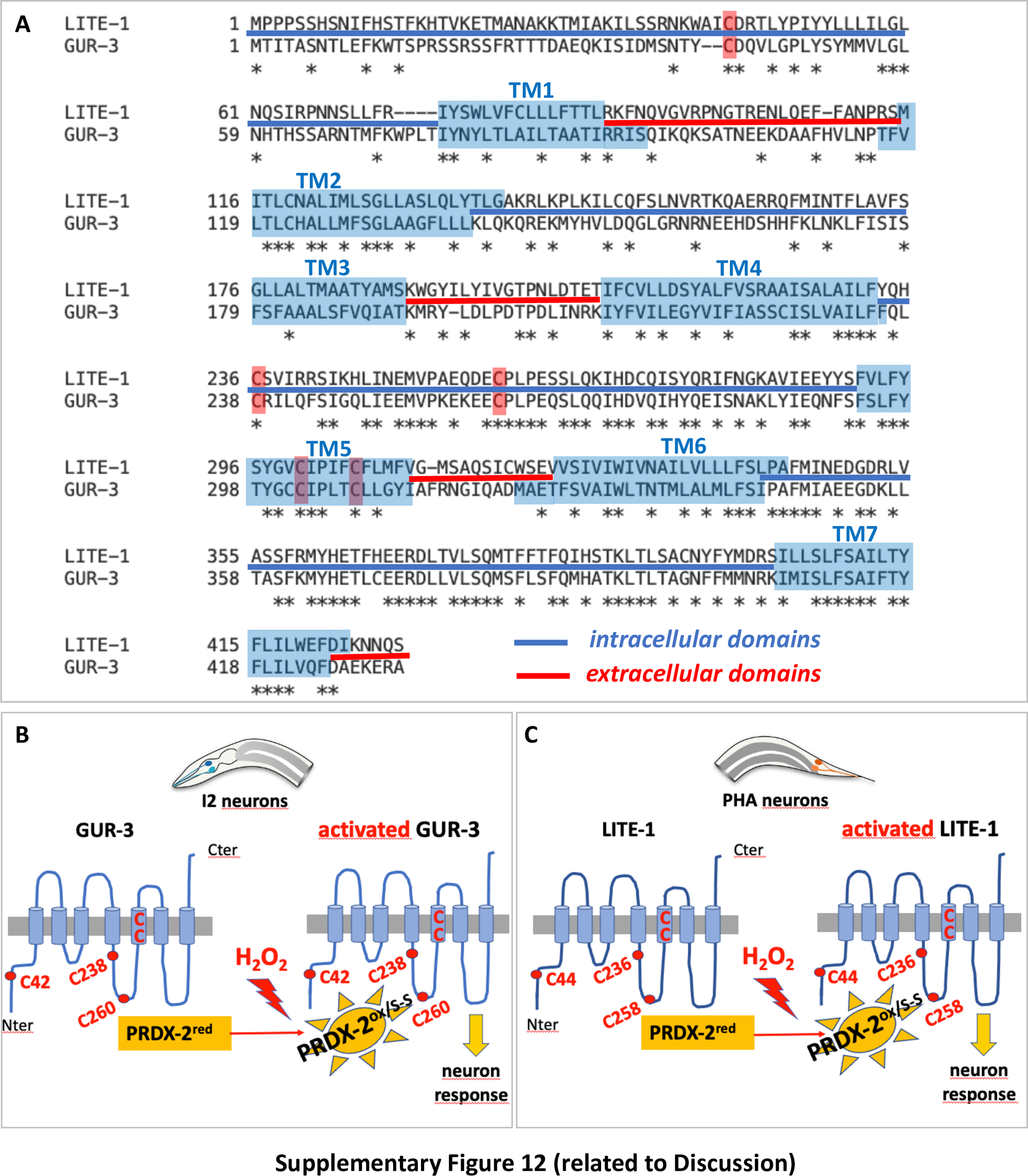

Supplement: S12 Fig — (A) Alignment of LITE-1 and GUR-3 protein sequences made with the SIM alignment tool (https://web.expasy.org/sim/), using the comparison matrix BLOSUM30. Transmembrane (TM) and intra/extracellular domains were predicted using the DeepTMHMM program (https://dtu.biolib.com/DeepTMHMM). The alignment reveals 39.9% identity over 434 residues overlap. Note the rather large intracellular domains (in blue), encompassing conserved cysteines (boxed in red) (B-C) A putative PRDX-2 redox relay may trigger H2O2-induced receptor activation in I2 and PHA neurons. Sketch of GUR-3 (B) and LITE-1 (C) receptors, deduced from A, depicting their conserved cysteines. Upon H2O2 exposure, oxidized PRDX-2 or its disulfide form (PRDX-2ox/S-S) could oxidize these cysteines, possibly forming a disulfide conjugate and/or inducing a conformation change, which would subsequently trigger receptor activation, and I2 or PHA neuron response. (TIF) [file pone.0274226.s012.tif]

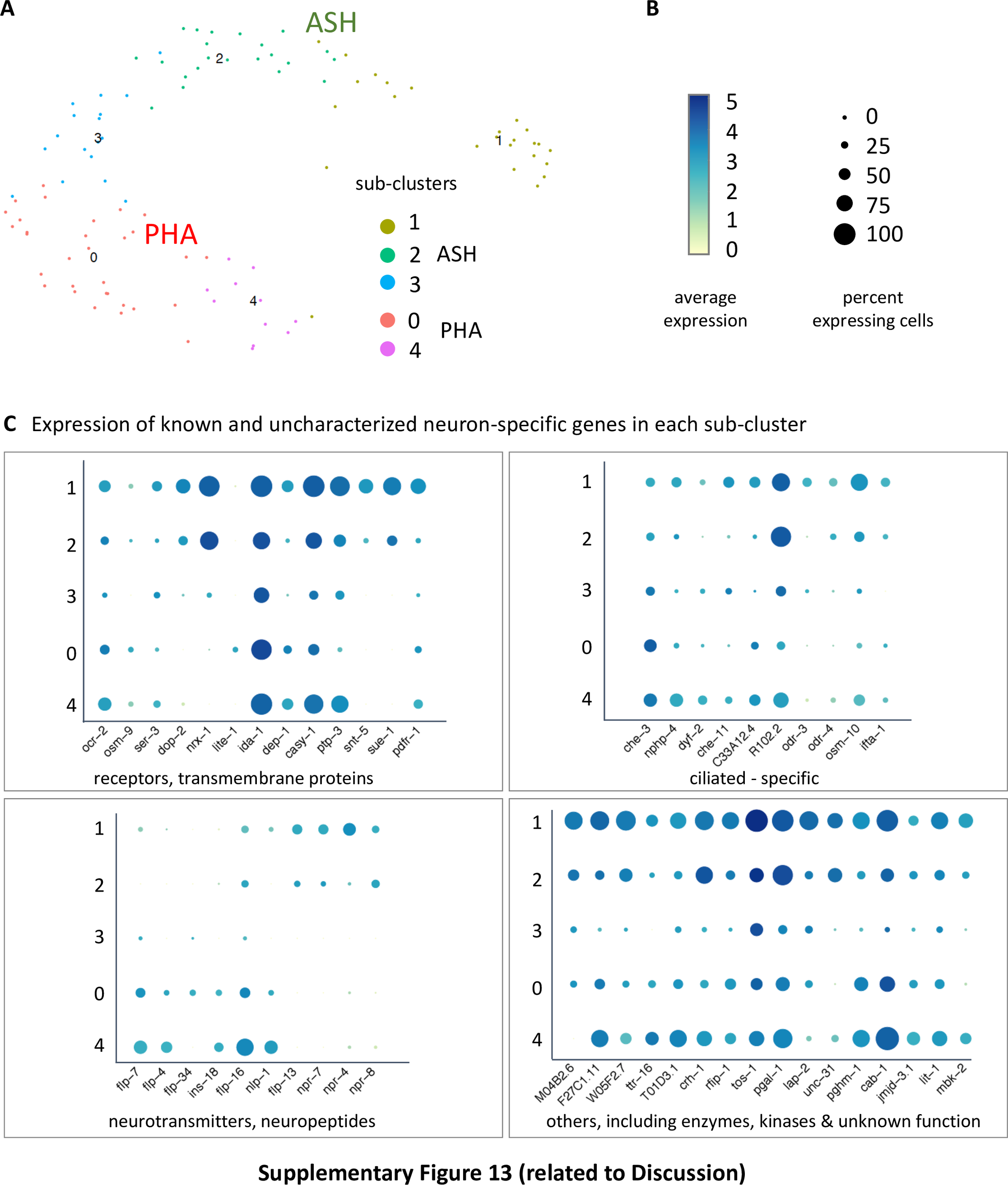

Supplement: S13 Fig — (A) Uniform Manifold Approximation and Projection (UMAP) projection of parent cluster 28, from [65] in which ASH and PHA/PHB nociceptive neurons were found in distinct sub-clusters (Louvain clustering at 8 PCs and at 1.2 resolution). (B, C) Dot plot indicating for a selection of genes both the intensity of gene expression and the fraction of expressing cells in each sub-cluster (B), based on single-cell RNA-sequencing data from [38]. See Supplementary Information and related S1–S3 Tables for exhaustive lists of genes expressed in each cluster. (TIF) [file pone.0274226.s013.tif]

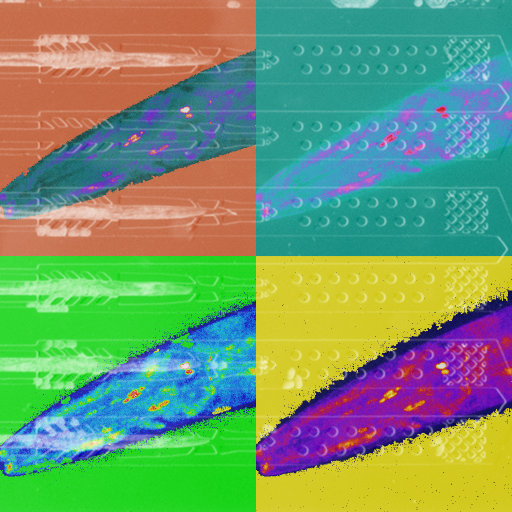

Supplement: S14 Fig — (TIF) [file pone.0274226.s014.tif]

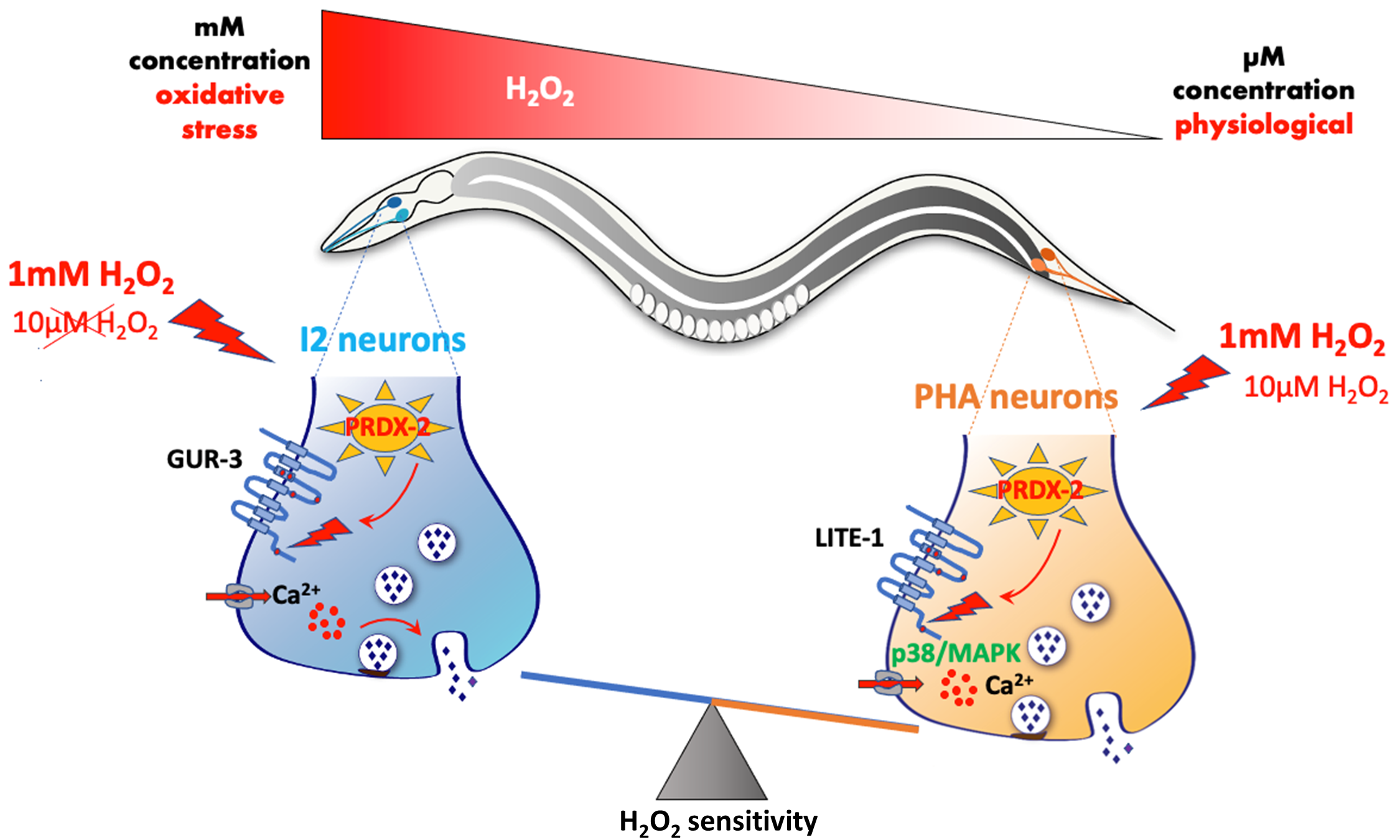

Supplement: S1 Graphical abstract — (TIF) [file pone.0274226.s039.tif]
