# Supplementary material for: Distinct mechanisms underlie H2O2 sensing in C. elegans head and tail
Source: PLoS One. 2022 Sep 29;17(9):e0274226. doi: 10.1371/journal.pone.0274226 (PMC9521893; doi:10.1371/journal.pone.0274226)
Supplement: S1 File — (PDF) [file pone.0274226.s040.pdf]

Supplementary Information

Quintin S., Aspert T., Ye T. and Charvin G., 2022

I- Matlab tutorial for analysis of *C. elegans* neuronal activation using a calcium sensor

These codes have been created for tracking timelapse recordings of left and right neuronal pairs expressing a calcium sensor such as GCaMP, and available at: https://github.com/gcharvin/viewworm. Below are described the sequential functions to run under Matlab to perform movie quantification.

Upon acquisition, movies should be cropped as small as possible to minimize processing time and should be save as .tif files.
Note: we recommend to first make a 4D-projection of the original movie, draw the smallest ROI around the neurons and transfer it to the original movie, to ensure that all frames are properly included in the cropped selection.

1. Movie registration

—> readworm_PHA ('movie_name.tif')

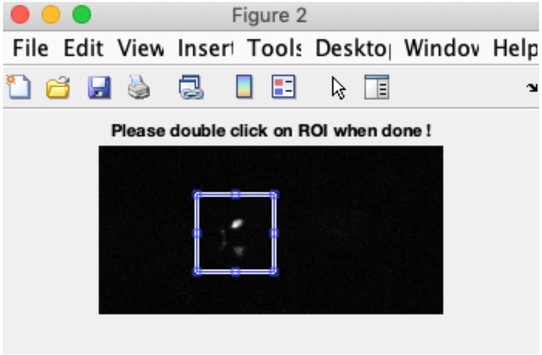

After ROI selection with the 2 neurons of interest (shown above), the function readworm_PHA processes every movie time point and performs the alignment of neurons, generating two files:

movie-registered.mat
movie.mp4

The mp4 allows the user to quickly verify that the movie has been correctly aligned.
Note: supplemental movies 1-14 were generated using this program.

2. Neuron segmentation, selection and tracking

—> viewworm_2('movie_name')

The function opens a figure project with several images and buttons.
The top left image allows movie traveling in the registered movie for each frame, using the right and left keyboard arrows, or typing directly the frame number in the box at the bottom of the window. To better visualize neurons, the intensity threshold of the image viewer can be adjusted with the top and bottom keyboard arrows.

Using the ‘**Pixel Train**’ button, the user teaches the program which pixels should be selected for neuronal segmentation by painting them in green (left mouse click). The pixels which should not be included for segmentation are painted in red (right mouse click). This needs to be done for several time points to obtain the best segmentation results, especially during the neuronal response. Below is shown an example with the selected neurons in green and the posterior neurite in red (excluded from the analysis), during the response.

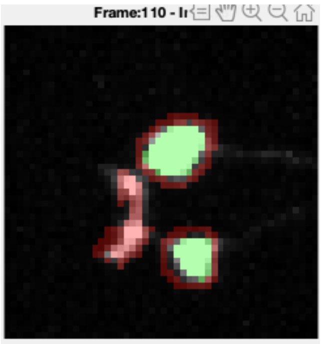

When this teaching has been done as often as needed, the user clicks ‘**Classify pixels**’, leading to the **neuron segmentation** as defined by the user. Then the user defines each neuron in the bottom segmented image, by clicking the ‘**Select neurons**‘ button:

right click—> red selection (left neuron);

left click —> blue neuron (right neuron);

Upon pressing the ‘**Track’** button, left and right neurons will be tracked for all movie time points. Upon pressing ‘**Plot’**, the **mean fluorescence intensity** is indicated as a function of time, generating the red and blue curves in the bottom right panel, as shown below.

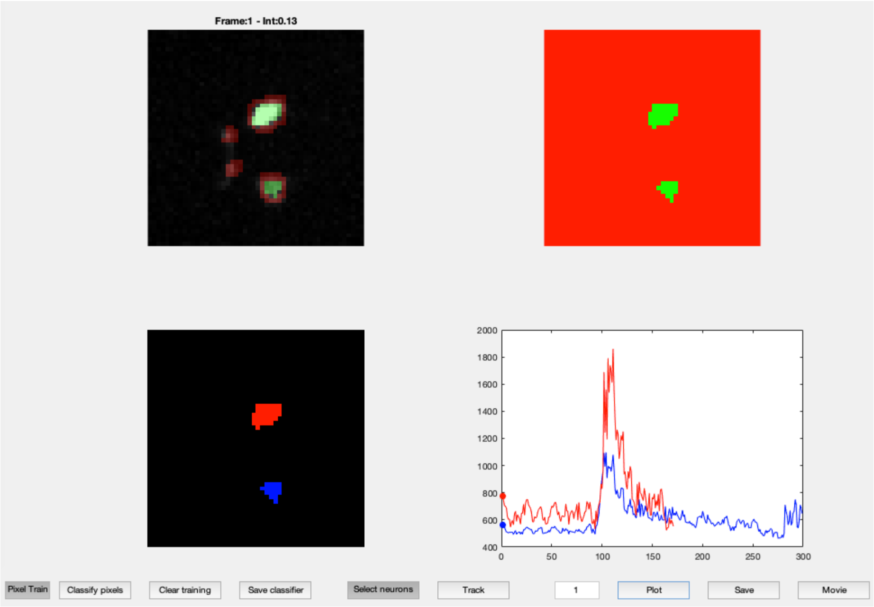

Important note : this step allows the **correction of tracking errors** at every frame. The quality control is performed by the user by selecting the appropriate neuron and clicking ‘**Track’** again until all tracking errors are corrected. Below is shown an example of error correction at time point 164 (pixels colored in green are excluded from the quantification in the bottom left box):

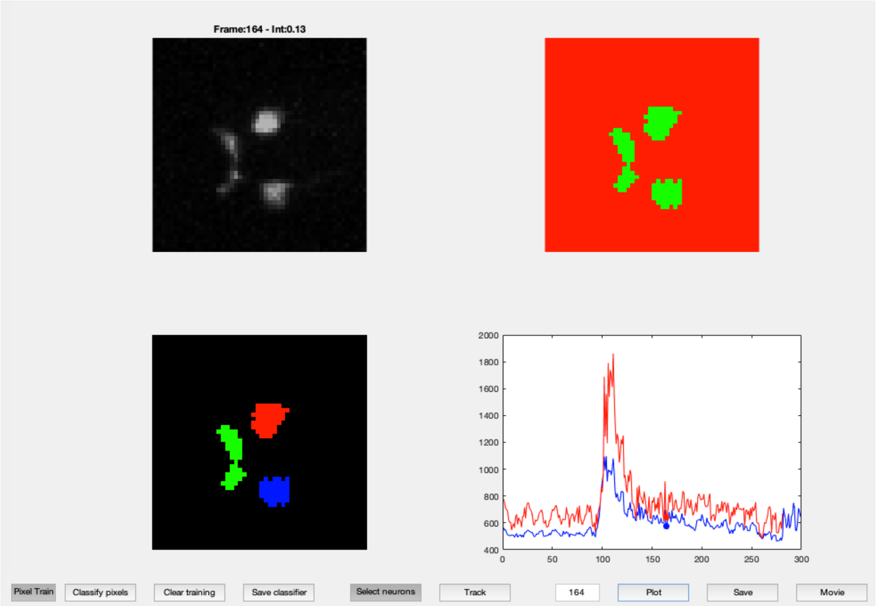

When the movie is properly tracked to the end, click '**Save'** button to obtain the .fig files which will be used for subsequent analyses. The user **needs to indicate the T0** in the command window, which corresponds to the time point at which the stress/ stimulus was applied.

Finally click on '**Movie'** to generate an mp4 movie showing the registered, segmented and tracked movies altogether, allowing an assessment of the global quality of the analysis.

Once all movies have been successfully processed, create a folder containing all the individual .fig analyzed files, which will be used for processing the average curve.

3. Average curve processing

—> averageGCamp2('folder_name')

In the command window, assign [low, high, offset]=averageGCamp2('folder_name')

—> processAverage('low,high,offset')

This function will generate average curves for red and blue neurons, for raw and normalized data.

Finally to retrieve individual intensity mean values at the response peak, run the **ExportSingleWormData** function which will produce an Excel sheet with all individual peak values.

II- Analysis of single cell transcriptomic data in ASH and PHA neurons (for the generation of the dot plots in S13 Fig)

Single-cell RNA-sequencing data was generated by (Cao et al. 2017). Clusters and sub-clusters were defined as described in (Lorenzo et al. 2020), using Louvain clustering at 8 PCs and at 1.2 resolution. The dataset normalization was performed using Seurat v4 (Hao et al. 2021), and visualized using dot plots. We performed differential analyses using the 'FindMarkers' function of Seurat v4 to compare cluster 28 versus all neurons (data in S1 Table); cluster 28.0 (PHA) versus all neurons (data in S2 Table); clusters 28.1 (ASH) versus all neurons (data in S3 Table). Selected genes were chosen among the most significantly enriched from these lists.

III- List of strains used

N2 wild type, reference strain
CTD1051.3 N2 *whEx127* [fNH059 (PRDX-2::GFP)+pRF4 *(rol-6(su1006)*] (Hirani et al. 2013)
SXB01 *prdx-2*(*gch01*[PRDX-2::GFP, *unc-119*(+)]) II; *unc-119(ed3)* III
SXB05 *prdx-2*(*gch03*[PRDX-2::GFP, *unc-119*(+)] II; *unc-119(ed3)* III
SXB15 5 times outcrossed SXB01 *prdx-2*(*gch01*[PRDX-2::GFP, *unc-119*(+)] II
SXB19 5 times outcrossed SXB05 *prdx-2*(*gch03*[PRDX-2::GFP, *unc-119*(+)] II
HT1593 7 times outcrossed *unc-119(ed3)* III (Dickinson et al. 2013)
QV225 *skn-1(zj15)* IV (Tang, Dodd, and Choe 2016)
SXB21 *prdx-2(gch03)* II; *skn-1(zj15)* IV
MT21650 *nIs575*[*flp-15*prom::GCaMP3, *lin-15*(+)] III; *lin-15(n765)* X (Bhatla and Horvitz 2015)
MT21570 *nIs575* III; *lite-1(ce314) lin-15(n765)* X (Bhatla and Horvitz 2015)
MT21785 *nIs575* III; *gur-3(ok2245) lin-15(n765)* X (Bhatla and Horvitz 2015)
KU4 *sek-1(km4)* IV (Kim et al. 2002)
KU25 *pmk-1(km25)* IV (Mizuno et al. 2004)
VC289 *prdx-2(gk169)* II (The *C. elegans* Deletion Mutant Consortium et al. 2012)
SXB53 *prdx-2(gk169)* II; *nIs575* III
SXB63 *unc-119(ed3)* III; *gur-3(gch16*[GUR-3::GFP, *unc-119*(+)]*)* X
SXB67 *nIs575* III; *pmk-1(km25)* IV
SXB70 *nIs575* III; *sek-1(km4)* X

All SXB strains were made in the course of this study.

IV- List of the oligonucleotides used to generate CRISPR/Cas 9 knock-in lines

All primers listed below are in the 5'-3'orientation.

1°) **PRDX-2::GFP knock-in**
Introduction of *prdx-2* sgRNA (into pMLS256):
Forward: **TTG**AGTGCTTCTTGAAGTACTCT
Reverse: **AAC**AGAGTACTTCAAGAAGCACT

PCR amplification of 5' and 3' homology arms (HAs) of *prdx-2* (including **SapI restriction** site and template-specific overhangs):

— 5' HA PCR product (542bp), upstream *prdx-2* stop codon:

Forward: TGT**GCTCTTC**TTggACCCACTTGACTTCACT
Reverse: gtg**GCTCTTC**gCGCGTGCTTCTTAAAGTATTCTTGACTTTCTTTGA (with silent mutations to change the PAM sequence)

— 3' HA PCR product (570bp), starting at *prdx-2* stop codon:

Forward: act**GCTCTTC**G**ggt**TAAatgtcttacatctctaatttcc
Reverse: tta**GCTCTTC**T**tac**atctccgtcctcctctaatgatgt

2°) **GUR-3::GFP knock-in**
Introduction of *gur-3* sgRNA :
Forward: **TTG**taagacaattttatTTACAC
Reverse: **AAC**GTGTAAataaaattgtctta

PCR amplification of 5' and 3' HAs of *gur-3* (with **SapI restriction** site and template-specific overhangs):

— 5' HA (614bp), upstream *gur-3* stop codon:

Forward: tga**GCTCTTC**a**TGG**gttatcatttaaccgtgattgcatgca
Reverse: gac**GCTCTTC**t**CGC**CACAGGTGGTTGGACAATGAGCA

— 3' HA (555bp), downstream *gur-3* stop codon:

Forward: tcc**GCTCTTC**t**GGT**TAAataaaattgtcttaacattttccccatattga
Reverse: ttt**GCTCTTC**g**TAC**tctcgtttaacgatttctcctgtctga

Supplementary References

Bhatla, Nikhil, and H. Robert Horvitz. 2015. “Light and Hydrogen Peroxide Inhibit *C. Elegans* Feeding through Gustatory Receptor Orthologs and Pharyngeal Neurons.” *Neuron* 85 (4): 804–18.

Cao, Junyue, Jonathan S. Packer, Vijay Ramani, Darren A. Cusanovich, Chau Huynh, Riza Daza, Xiaojie Qiu, et al. 2017. “Comprehensive Single-Cell Transcriptional Profiling of a Multicellular Organism.” *Science* 357 (6352): 661–67.

Consortium, The *C. Elegans* Deletion Mutant, and The *C. elegans* Deletion Mutant Consortium. 2012. “Large-Scale Screening for Targeted Knockouts in the *Caenorhabditis Elegans* Genome.” *G3 Genes|Genomes|Genetics*. https://doi.org/10.1534/g3.112.003830.

Dickinson, D. J., J. D. Ward, D. J. Reiner, and B. Goldstein. 2013. “Engineering the *Caenorhabditis Elegans* Genome Using Cas9-Triggered Homologous Recombination.” *Nature Methods*. https://www.nature.com/nmeth/journal/v10/n10/abs/nmeth.2641.html.

Hao, Yuhan, Stephanie Hao, Erica Andersen-Nissen, William M. Mauck 3rd, Shiwei Zheng, Andrew Butler, Maddie J. Lee, et al. 2021. “Integrated Analysis of Multimodal Single-Cell Data.” *Cell* 184 (13): 3573-3587.e29.

Hirani, Nisha, Marcel Westenberg, Minaxi S. Gami, Paul Davis, Ian A. Hope, and Colin T. Dolphin. 2013. “A Simplified Counter-Selection Recombineering Protocol for Creating Fluorescent Protein Reporter Constructs Directly from *C. Elegans* Fosmid Genomic Clones.” *BMC Biotechnology* 13 (January): 1.

Kim, Dennis H., Rhonda Feinbaum, Geneviève Alloing, Fred E. Emerson, Danielle A. Garsin, Hideki Inoue, Miho Tanaka-Hino, et al. 2002. “A Conserved P38 MAP Kinase Pathway in *Caenorhabditis Elegans* Innate Immunity.” *Science* 297 (5581): 623–26.

Lorenzo, Ramiro, Michiho Onizuka, Matthieu Defrance, and Patrick Laurent. 2020. “Combining Single-Cell RNA-Sequencing with a Molecular Atlas Unveils New Markers for *Caenorhabditis Elegans* Neuron Classes.” *Nucleic Acids Research* 48 (13): 7119–34.

Mizuno, Tomoaki, Naoki Hisamoto, Takashi Terada, Tae Kondo, Makoto Adachi, Eisuke Nishida, Dennis H. Kim, Frederick M. Ausubel, and Kunihiro Matsumoto. 2004. “The *Caenorhabditis Elegans* MAPK Phosphatase VHP-1 Mediates a Novel JNK-like Signaling Pathway in Stress Response.” *The EMBO Journal* 23 (11): 2226–34.

Niu, Wei, Zhi John Lu, Mei Zhong, Mihail Sarov, John I. Murray, Cathleen M. Brdlik, Judith Janette, et al. 2011. “Diverse Transcription Factor Binding Features Revealed by Genome-Wide ChIP-Seq in *C. Elegans*.” *Genome Research* 21 (2): 245–54.

Tang, Lanlan, William Dodd, and Keith Choe. 2016. “Isolation of a Hypomorphic *Skn-1* Allele That Does Not Require a Balancer for Maintenance.” *G3 Genes|Genomes|Genetics* 6 (3): 551–58.
